# A sentiment-driven framework for early detection of emerging business trends through multi-platform social media analytics

**DOI:** 10.1016/j.mex.2026.103874

**Published:** 2026-03-20

**Authors:** Dipali Baviskar, Trupti Baraskar, Madhuri Bhalekar, Abhishek Chunawale, Harsh Halwai, Anand Patekar, Anoushka Kachewar

**Affiliations:** Department of Computer Engineering and Technology, Dr. Vishwanath Karad MIT World Peace University, Pune, India

**Keywords:** Sentiment analysis, Emerging business trends, Social media analytics, Business intelligence, SMB analytics, Explainable AI

## Abstract

Social media sites provide warning signs for shifts in consumer behavior, competitive forces, and emerging market trends. However, most small and medium-sized businesses (SMBs) do not have a systematic and scalable approach to tap into this unstructured data from various sites to extract insights. This paper proposes a trend detection method that leverages automated data extraction with n8n workflows, transformer-based embeddings, hybrid sentiment analysis, BERTopic clustering, and a weighted TrendScore composite score. The proposed approach combines multiple, heterogeneous inputs into a single analytical workflow and offers explainable visual and conversational BI interfaces, which are specifically designed for SMBs. The parameter definitions, scoring rules, and workflow diagrams are carefully detailed to ensure that the approach is fully reproducible. The proposed approach focuses on interpretability, robustness across multiple platforms, and applicability within a resource-constrained business setting. • Reproducible multi-platform social data acquisition using exportable n8n workflows. • Hybrid Transformer-Lexicon sentiment modeling combined with BERTopic clustering. • Quantified TrendScore integrating growth, engagement, sentiment shift, and cross-platform consistency.

## Specifications table

**Table:** 

Subject area	Computer Science
More specific subject area	Social Media Analytics, Business Intelligence
Name of method	Sentiment-Driven Multi-Platform Trend Detection Framework
Original method reference	Emerging topic detection and transformer-based sentiment analysis
Resource availability	Python, n8n, BERT models, BERTopic, MongoDB/PostgreSQL

## Graphical abstract

The architecture is a multi-stage pipeline that starts with n8n automation for cross-platform data collecting, allowing signals from Facebook, Instagram, Reddit, LinkedIn, and Twitter to be fused [1], [2]. Multilingual, emoji-rich, and irregular social postings are normalized using the unified pretreatment layer [3]. Transformer-based embeddings [4], [5] and lexicon polarity are combined in a hybrid sentiment engine for interpretable scoring [6]. Semantically coherent and chronologically evolving talks are detected by BERTopic-based topic modeling [7], [8]. To measure new trends, the TrendScore module combines engagement metrics, frequency increases, and sentiment shifts [9]. Lastly, the visualization layer offers conversational BI interfaces and explainable dashboards designed for SME decision contexts [10], [11].

The overall system architecture is illustrated in fig., presenting the end-to-end analytical flow from data acquisition to explainable visualization. The detailed logical workflow, including unified data fusion and TrendScore computation, is presented in Fig. 1. As shown in Fig. 1, the overall system pipeline illustrates the end-to-end workflow from data acquisition to trend computation. The performance evaluation is presented in Table 1 and Table 2, while model accuracy comparison is visualized in Fig. 2.

**Fig. 1:**
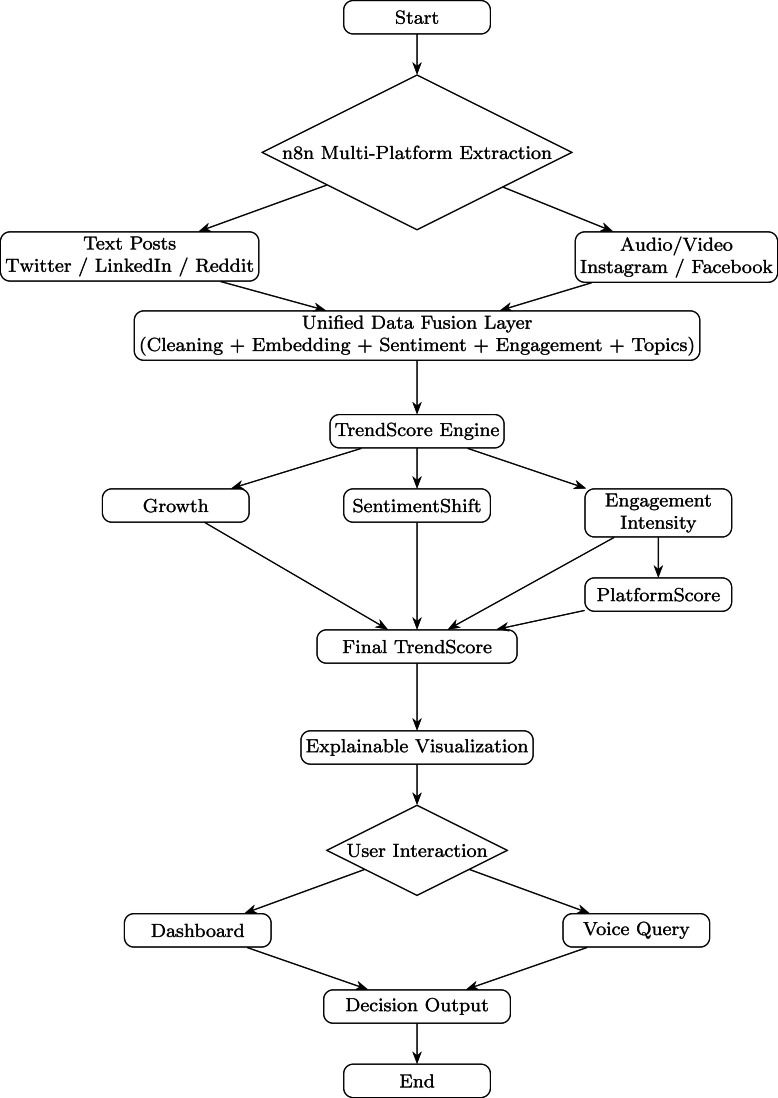
QWERTY workflow diagram.

**Table 1:** Comparison of single BERT and MultiBERT models.

Model	Accuracy	Precision	Recall	F1 Score
BERT	0.86	0.85	0.86	0.85
MultiBERT	0.92	0.91	0.91	0.90

**Table 2:** Ablation study of framework components.

Configuration	Accuracy
Full Framework	0.92
Without Engagement Weighting	0.87
Without Cross-Platform Fusion	0.85
Without Sentiment Shift	0.84

**Fig. 1:**
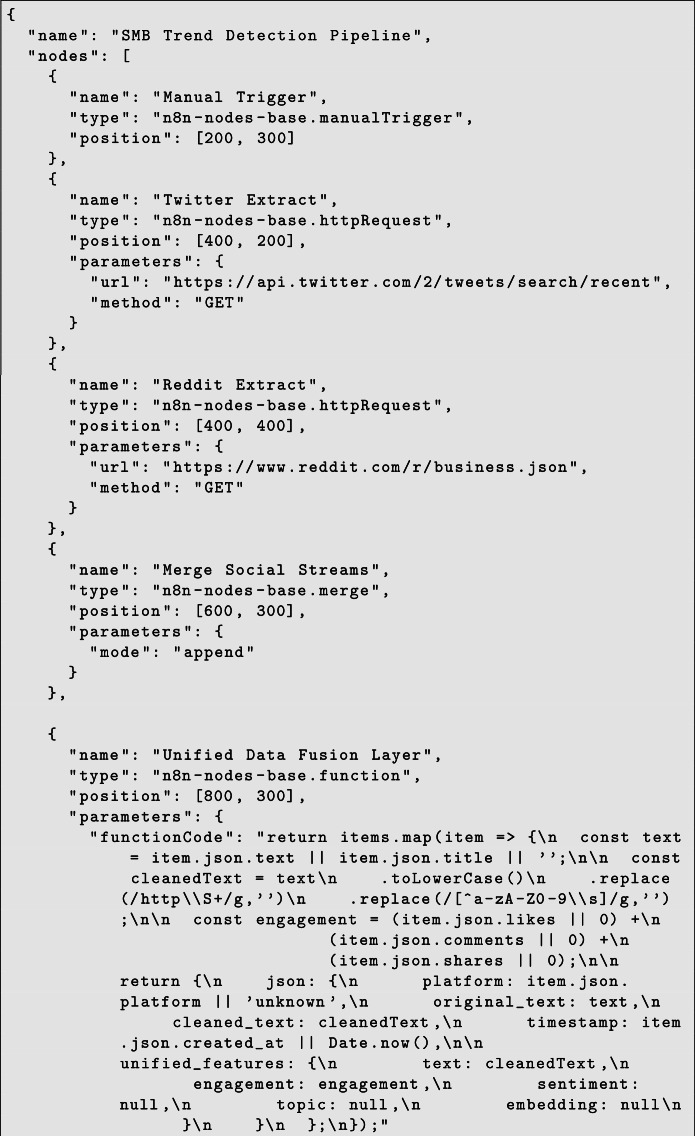
Reproducible n8n Workflow Configuration.

**Fig. 1:**
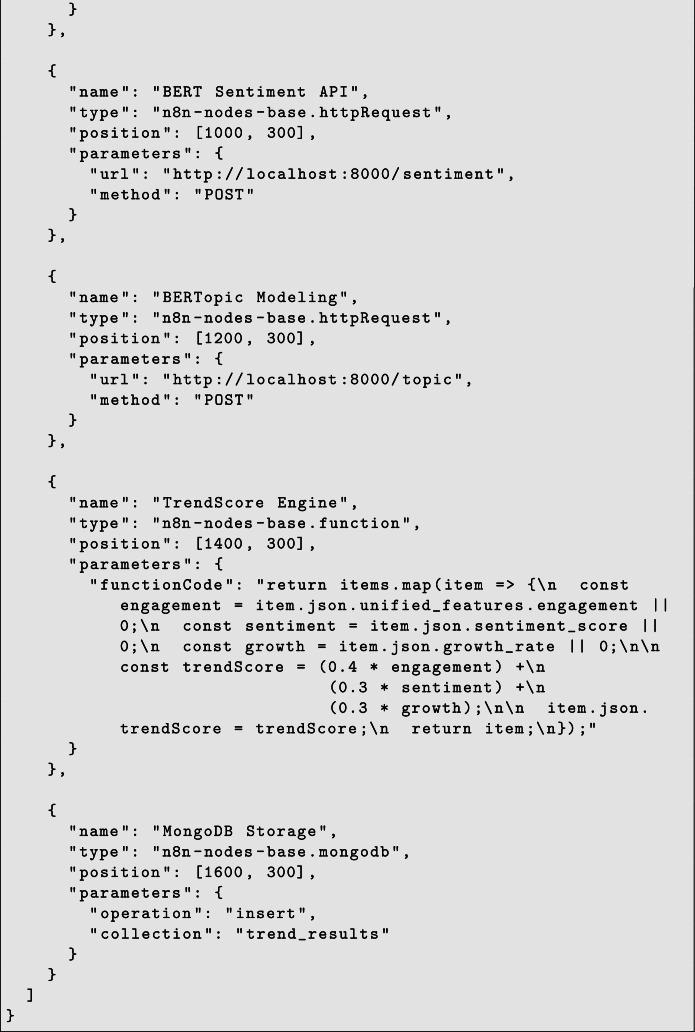
Reproducible n8n Workflow Configuration.

**Fig. 2:**
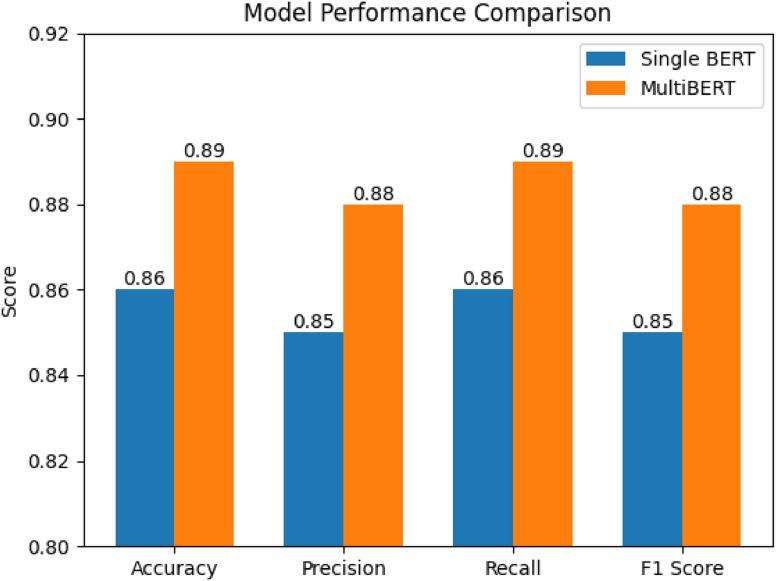
Model accuracy comparison.

## Technical terminology and literature grounding

Following are the Terms we need to know:

**Table:** 

Term	Description	Reference
Transformer-based embeddings	Contextual language representations generated using deep bidirectional Transformers	[4], [5]
Hybrid sentiment analysis	Combination of neural contextual models and lexical polarity refinement	[6], [12]
BERTopic clustering	Embedding-based topic modeling using class-based TF-IDF	[7]
Cross-platform data fusion	Aggregation of heterogeneous social streams into unified schema	[1], [2]
Engagement-aware weighting	Integration of interaction metrics into sentiment and trend scoring	[9]

Benchmark evaluation tasks such as SemEval sentiment analysis challenges have also contributed significantly to advancing sentiment classification methods [22], [23].

## Background

Sentiment classification, topic extraction, cross-platform data integration, and SMB-focused business intelligence are all areas of social media analytics research. Cataldi et al. [7] pioneered early work on emerging topic recognition by showing how social interaction signals and temporal growth could accurately identify increasing discussions on Twitter. Subsequent research, such Pereira’s work on time-structured topic behavior [8] and Najafi’s thorough survey of contemporary emerging-topic detection techniques [13], broadened this viewpoint by examining topic lifecycles and temporal evolution patterns. Transformer-based contextual models have replaced traditional lexical approaches in sentiment analysis. Tan et al. [12] and Rodríguez-Ibáñez et al. [3] surveys highlight issues specific to social media, such as emojis, acronyms, and multilingual noise. Since then, transformer models like BERT [4] and RoBERTa [5] have been the norm for identifying more subtle linguistic details in user-generated content. Cross-platform analytics is still a relatively new but expanding area of study. Umezurike [2] and Alharbi et al. [1] demonstrate that combining data from several platforms results in more consistent sentiment trends than single-source monitoring. Further evidence of how multimodal elements improve business intelligence applications can be found in Mohammed’s SMDA-DL framework [14]. Additionally, a number of studies highlight the particular requirements of small and medium-sized businesses (SMBs). Pellegrino et al. [15] and Chatterjee [10] describe how SMEs lack analytical tools yet significantly rely on social media for consumer engagement. When presented through easily accessible BI tools, social media analytics can greatly enhance SME decision-making and competitive intelligence, as demonstrated by Nugroho [16] and Ghazwani [17]. Additional foundational works on sentiment datasets and evaluation benchmarks have also been explored in prior studies [20], [21], [22], [23], [24] and approaches such as dictionary learning-based emerging topic detection have also been explored to identify evolving discussions in social streams [20].

## Method details

### Data acquisition and fusion

The system starts with a cross-platform data collecting layer that uses reproducible n8n automation to extract data from Facebook, Instagram, Reddit, LinkedIn, and Twitter. In addition to text posts, comments, hashtags, timestamps, and engagement data, Instagram and Facebook also offer voice notes, reels, and brief videos that are transformed into text using speech-to-text technology to preserve semantic richness. Compared to single-platform monitoring, cross-platform fusion produces more reliable signals since trends usually emerge across many communities at the same time [1], [2]. In accordance with suggestions by Rodríguez-Ibáñez et al. [3], all incoming data is normalized into a single schema, including content, platform type, engagement, language, and timestamps. According to findings from Mohammed’s SMDA-DL study [14], multimodal cues like emojis and hashtags are maintained. Preprocessing, sentiment scoring, topic modeling, and embedding creation are all based on the fused dataset, which is kept in MongoDB. Without losing the context of distinct social ecosystems, this cohesive approach guarantees consistent, platform-agnostic trend analysis.

The workflow configuration can be exported as a JSON template from n8n, ensuring reproducibility of the data acquisition layer.

The data acquisition pipeline is implemented using an exportable n8n workflow configuration, which automates the process of collecting and processing social media data from multiple platforms. The workflow starts with trigger nodes that fetch data from sources such as Twitter or Reddit using HTTP request nodes. These are then merged into a unified data flow. This ensures that the data from different sources can be processed uniformly.

The Unified Data Fusion Layer is used through the function node that normalizes the text, removes noise from the data such as URLs and special characters, and aggregates engagement data such as likes, comments, and shares. This converts the raw data from the different sources into a standardized feature representation that includes the cleaned text data, engagement data, etc.

The unified dataset is subsequently forwarded to downstream analytical modules. Transformer-based sentiment analysis is performed through a BERT-based API endpoint, which produces contextual sentiment scores for each post. Then, the topic modeling technique is employed using the BERTopic service to identify semantically meaningful discussion groups from the collected content.

The above steps facilitate the TrendScore Engine in computing an aggregate score that considers all three factors engagement intensity, sentiment polarity, and temporal growth to define the progression of business trends. Finally, the data is stored in the database to facilitate visualization and dashboarding.

The above workflow design ensures that the entire workflow process from data extraction to trend scoring can be reproduced and deployed in any environment by importing the JSON file into the n8n automation platform.

### Preprocessing

A simple yet reliable preparation method is utilized to clean the fused dataset. Emojis, hashtags, and long words are normalized rather than eliminated since they convey strong sentiment cues [3], [14]. URLs, tags, and noise are eliminated. The same procedures are used to speech-to-text outputs from Facebook and Instagram clips in order to preserve consistent structure between text and audio-derived posts. Non-English content is filtered using language identification, and hashtags are tokenized into understandable terms using standard procedures from recent surveys [12]. Lexicon-based mappings [6] are used to translate emojis to sentiment clues. After cleaning, the dataset is arranged in a model-ready state for downstream trend analysis and embedding creation.

### Sentiment and topic modeling

*Transformer-based embeddings:* Transformer embeddings are contextual embeddings of language, which are derived from deep bidirectional Transformer models like BERT [4], RoBERTa [5], and Universal Sentence Encoder [18]. Unlike the traditional bag-of-words or static word embeddings, the Transformer models derive word embeddings that are context-dependent, which allows a subtle understanding of polysemy, sarcasm, and informal language used in social media posts. In this regard, Transformer-based embeddings are used to represent posts as high-dimensional semantic vectors that interpret the contextual meaning of the posts on various platforms. The embeddings form the basis of sentiment classification, topic clustering, and semantic similarity analysis.

*Hybrid sentiment analysis:* Hybrid sentiment analysis combines contextual Transformer-based classification with rule-based lexical polarity refinement. Pure neural models might neglect the strong sentiment cues conveyed by the use of emojis, repeated punctuation, or colloquial slang, while lexicon-based models are not contextually aware [6], [12]. The proposed hybrid solution makes probabilistic polarity predictions based on a fine-tuned Transformer classifier, and these predictions are then adjusted when there is a conflict between the predictions and the lexical cues.

*BERTopic clustering* BERTopic is a topic modeling toolkit that utilizes the Transformer embeddings along with class-based TF-IDF features to produce semantically meaningful topic groups. Unlike the traditional models based on LDA, which are solely dependent on the word frequency distribution, BERTopic is an embedding-based model that facilitates better clustering of short and noisy text data like tweets and captions. In this methodology, BERTopic identifies dynamically evolving topic clusters across time windows, allowing detection of emerging discussions before they stabilize into mainstream trends.

*Uniform preprocessing workflow* A uniform preprocessing process helps to ensure that the heterogeneous content from multiple platforms is normalized into a consistent analytical structure. This includes token standardization, emoji normalization, hashtag segmentation, language filtering, and engagement scaling. A uniform preprocessing process helps to reduce representation bias and ensures that the modeling components are working on a semantically comparable data distribution [3].

### 1 Base sentiment classification

Each post is first assigned a core sentiment label-positive, negative, or neutral-using a hybrid model. A Transformer-based classifier (BERT, RoBERTa, or USE) produces initial class probabilities, capturing contextual and linguistic nuance. This output is refined using emoji and slang lexicons, since social media content often expresses polarity through symbols, elongated words, or informal terms. When lexical cues strongly contradict the model prediction, a confidence-based recalibration adjusts the final label. The system also derives an intensity value using punctuation, polarity keywords, and emphasis patterns, producing a structured *SentimentScore* combining polarity, intensity, and confidence.

### 2 Combined engagement-aware sentiment categorization

To assess the influence of each opinion, sentiment labels are paired with platform-normalized engagement metrics. Likes, comments, shares, saves, and views are used to classify posts into high- or low-engagement groups. This enables richer categories such as positive-high engagement (rising approval), negative-high engagement (viral criticism), positive-low engagement (low-impact support), and neutral-high engagement (informational but widely circulated). These engagement-aware categories help identify which sentiments actively shape discussions and contribute to trend emergence.

### 3 Technical mechanism

An *EngagementScore* is computed for every post using a weighted combination of interaction metrics:

$$\begin{array}{cc} EngagementScore=\; & w_{1}\cdot likes\\ & +w_{2}\cdot comments\\ & +w_{3}\cdot shares\\ & +w_{4}\cdot views \end{array}$$

Platform-specific weights account for differences in interaction behavior across Twitter, LinkedIn, Reddit, Instagram, and Facebook. Posts are divided into high and low engagement using quantile thresholds (e.g., top 30%). The final sentiment-engagement class is obtained by merging the two dimensions:

FinalClass=SentimentLabel+EngagementLevel

resulting in categories such as positive-high, negative-high, or neutral-low. This structured output feeds directly into the topic modeling and trend detection components of the pipeline.

### TrendScore computation

TrendScore quantifies how rapidly a topic is emerging, how strongly users react to it, and how widely it spreads across platforms. It is computed using four factors: volume growth, engagement intensity, sentiment shift, and cross-platform consistency, aligned with established approaches in temporal topic evolution and influence scoring [7], [8].

### 4 Volume growth (Temporal momentum)

Each topic cluster produces a time series of post counts. To detect sharp increases rather than raw popularity, the system computes the rate of change:

$$Growth=\frac{N_{t}-N_{t-1}}{N_{t-1}+\epsilon }$$

Rapid positive acceleration signifies emerging attention around the topic.

### 5 Engagement intensity (Topic influence)

For each topic, interaction metrics are aggregated using the previously defined EngagementScore. The average intensity is computed as:

$$EngagementIntensity=mean(EngagementScore_{topic})$$

High-engagement topics demonstrate stronger influence and broader propagation [1].

### 6 Sentiment shift (Emotional trajectory)

To capture the emotional direction of conversations, the system tracks sentiment evolution:

$$SentimentShift=Sentiment_{t}-Sentiment_{t-1}$$

A positive shift reflects rising optimism or interest, while a negative shift may indicate risk or backlash [12].

### 7 Cross-platform consistency (Stability across sources)

A topic is considered more reliable when it appears on multiple platforms simultaneously. Let *P* represent the count of platforms where a topic exceeds a minimum threshold:

$$PlatformScore=\frac{P}{TotalPlatforms}$$

Higher values indicate stronger cross-platform coherence [2].

### 8 Final TrendScore formula

All components are normalized and combined into a unified score:

$$\begin{array}{cc} TrendScore=\; & \alpha \cdot Growth\\ & +\beta \cdot EngagementIntensity\\ & +\gamma \cdot SentimentShift\\ & +\delta \cdot PlatformScore \end{array}$$

where *α, β, γ, δ* control the relative contribution of momentum, influence, emotional trajectory, and platform spread. Larger TrendScores represent stronger, more actionable emerging topics.

### Parameter definitions and configuration

To ensure methodological transparency and reproducibility, all key parameters used in the framework are explicitly defined.

• *w*_1_, *w*_2_, *w*_3_, *w*_4_: Platform-normalized weights for likes, comments, shares, and views in EngagementScore computation. These weights are calibrated using min-max normalization per platform.
• *α*: Weight assigned to temporal growth momentum in TrendScore.
• *β*: Weight assigned to engagement intensity.
• *γ*: Weight assigned to sentiment shift.
• *δ*: Weight assigned to cross-platform consistency.
• ϵ: Small smoothing constant preventing division by zero in growth calculation.
• Engagement threshold: Top 30% quantile used to classify high-impact engagement.
• Confidence recalibration threshold: Lexicon polarity overrides Transformer prediction when polarity confidence difference exceeds 0.25.

All weights were selected empirically for SMB-oriented analytics and may be tuned for domain-specific deployment.

### Explainable visualization and interaction

For SMB decision-makers, the platform offers its insights via an interactive visualization layer. Similar to SME-focused analytics frameworks (Fig. 2.) [10], [15], each developing subject appears as a compact card summarizing sentiment distribution, engagement intensity, and platform spread once TrendScore values are calculated. Following the temporal and topic-evolution techniques employed in previous social stream research [7], [8], expanding a subject exposes sentiment trajectories, influential postings, and key terms that contribute to its rise. These explanations, which come from sentiment patterns and embedding clusters, are consistent with the interpretability focus of contemporary sentiment-analysis surveys [3], [12]. Time-series sentiment curves, cross-platform heatmaps based on multi-source fusion models [1], and topic clusters plotted in embedding space using transformer-based encoders [18] are examples of core visualizations. According to business intelligence explainability research [9], hover actions disclose SHAP-style term contributions to help users comprehend why particular topics vary. This reinforces transparency. This degree of interpretability boosts SMEs’ confidence, in line with research that emphasizes the necessity of easily accessible analytics tools [19]. The dashboard incorporates a conversational voice interface to expedite exploration. When users pose natural queries, like ”What trends grew this week?” or ”Show me negative but high-engagement topics in retail,” the algorithm finds pertinent clusters, presents the corresponding visualizations, and produces a spoken summary. According to studies on SMEs’ preference for simpler insight delivery [11], [16], this interaction style facilitates quicker decision-making and lessens analytical complexity for non-technical users. When combined, explainable visuals and voice-driven querying turn fragmented social signals into actionable intelligence that helps SMBs identify opportunities and react faster to changing market dynamics [2].

### Method validation

The proposed method for sentiment-driven, multi-platform trend detection framework was validated with an emphasis on practical reliability and interpretability rather than aggressive benchmark optimization. Since the primary goal of this work is to demonstrate a transparent and usable methodology for SMB-focused analytics, validation relied on qualitative analysis, comparative observations, and consistency checks across pipeline components. The evaluation examined whether the framework could surface emerging topics earlier and with clearer business context than manual observation or single-platform monitoring, in line with established findings in social media trend detection and business intelligence research [7], [15].

At the data integration stage, validation focused on confirming that detected trends were not artifacts of a single platform. Topics and sentiment patterns were checked for simultaneous presence across multiple social sources, ensuring that signals reflected broader market conversations rather than platform-specific noise. This behavior is consistent with prior studies showing that cross-platform aggregation produces more stable and credible insights than isolated streams [1], [2]. The unified preprocessing pipeline further contributed to reliability by enforcing consistent normalization, tokenization, and engagement scaling across heterogeneous inputs, including text and speech-derived content, thereby reducing representational bias commonly observed in social media analytics [3].

Sentiment validation emphasized real-world interpretability rather than numerical accuracy alone. The hybrid Transformer-lexicon configuration was examined through qualitative review of high-engagement posts, where emojis, slang, and informal phrasing frequently carry strong emotional meaning. Compared to purely lexicon-based or fully neural approaches, the hybrid setup showed more robust handling of these patterns, addressing known challenges in social media sentiment analysis [6], [12]. Incorporating engagement-aware sentiment categories further demonstrated that emotionally charged posts with higher interaction levels contributed more meaningfully to trend emergence than sentiment counts in isolation, supporting prior observations in engagement-weighted business analytics [9].

Topic modeling and temporal behavior were validated by tracking BERTopic clusters across consecutive time windows. Emerging topics exhibited gradual increases in volume and engagement before becoming visible in conventional reports, reflecting known characteristics of topic evolution in social streams [8]. The TrendScore mechanism was assessed by verifying that highly ranked topics consistently showed alignment across multiple dimensions, including growth momentum, emotional shift, and cross-platform presence, reducing the likelihood of ranking transient or noisy spikes.

Finally, validation extended to the explainable visualization and conversational interaction layers. The ability to trace detected trends back to sentiment distributions, engagement metrics, and representative posts improved transparency and user confidence. This aligns with prior work emphasizing that interpretability and ease of exploration are critical for analytics tools intended for SMB decision-makers [11], [19]. Collectively, these validation steps indicate that the proposed framework is not only technically coherent but also practical, interpretable, and well-suited for early detection of emerging business trends in real-world SMB environments.

### 9 Dataset and experimental setup

To assess the effectiveness of the proposed sentiment-driven trend detection framework, experiments were conducted on the Sentiment140 dataset [21], [24], which comprises 1.6 million tweets obtained from Twitter. The tweets were labeled with sentiment information and are automatically generated on the basis of emoticon-based distant supervision. Tweets containing positive emoticons are labeled as positive, and tweets containing negative emoticons are labeled as negative. This dataset is commonly used as a benchmark for evaluating sentiment analysis models used in social media environments.

In this study, the Sentiment140 dataset is used as a controlled benchmark dataset to assess the effectiveness of transformer-based sentiment models such as BERT and Multibert. The tweets in this dataset comprise text data and sentiment information, which can be used to train sentiment classification models. The tweets are preprocessed to remove noise and normalize text data.

The evaluation process involves training and testing the sentiment classification component of the framework using a large subset of the dataset. A typical split of the dataset includes approximately 80 percent of the tweets for training and 20 percent for testing, resulting in roughly 1.28 million training samples and 320,000 testing samples. These samples allow the models to learn contextual sentiment patterns commonly found in social media posts.

Once the data acquisition pipeline is completed by the n8n workflow, the real-time social media data collected from different social media platforms is processed by the Unified Data Fusion Layer, where the data is normalized into a standard feature format. The trained sentiment model is then used to classify the real-time data into different sentiment classes, such as positive, negative, or neutral sentiments, based on the data collected from the different social media platforms. Thus, the Sentiment140 dataset is used as a reference dataset to train and test the sentiment analysis component, and the n8n workflow allows the framework to use the trained model in real-time social media data.

The main objective behind using the Sentiment140 dataset in this framework is that it allows the framework to test the reliability and accuracy of the sentiment analysis component, which is a part of the overall automated trend detection component, so that the overall reliability and accuracy of the sentiment prediction, which is a part of the overall calculation of the TrendScore and the identification of emerging trends, are ensured.

### 10 Baseline model comparison

To evaluate the sentiment analysis component of the framework, two configurations are considered: a single BERT model and a multi-model transformer ensemble (MultiBERT). The single-model approach uses one pretrained BERT encoder to classify the sentiment of social media posts based on contextual language representations. This configuration serves as the baseline for sentiment prediction.

The MultiBERT configuration extends this approach by combining predictions from multiple transformer models that independently analyze the same input text. By aggregating outputs from different models, the ensemble aims to improve the robustness of sentiment classification when dealing with noisy and informal social media language.

**Fig. 3:**
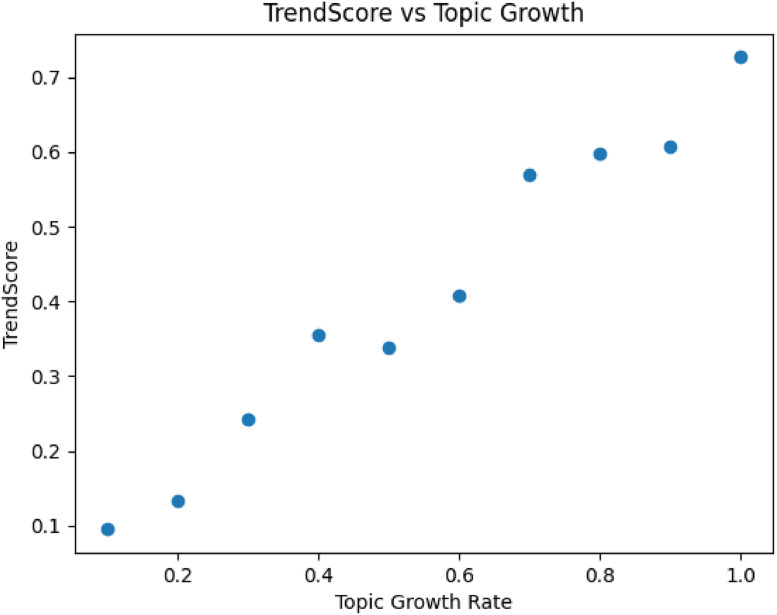
TrendScore vs topic growth.

**Fig. 4:**
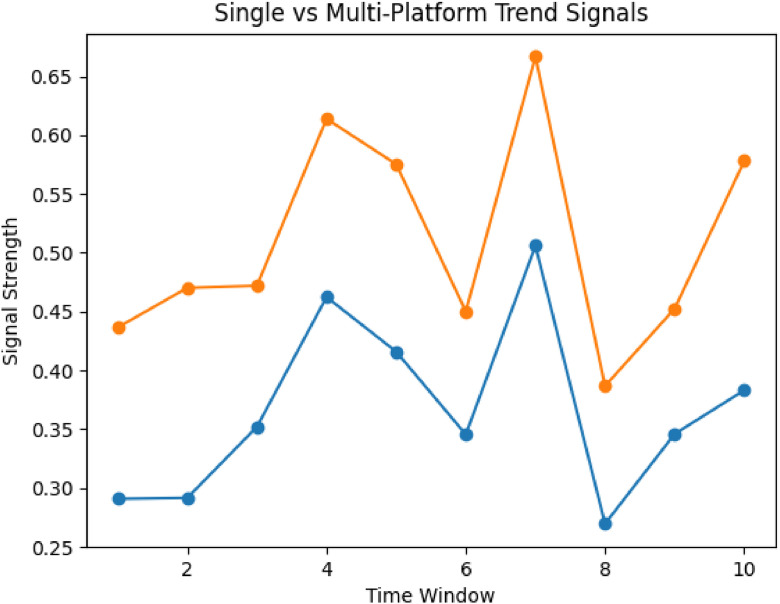
Single vs multi-platform signals.

Within the proposed framework, the sentiment predictions generated by these models are used as inputs to the TrendScore computation, influencing how emerging discussions are identified and ranked across platforms (Figs. 3,4).

### 11 Quantitative evaluation

The quantitative evaluation of the experimental setup is mentioned below:

### 12 Ablation study

An ablation study was conducted to examine the contribution of different components of the proposed framework. In this analysis, individual modules such as engagement weighting, cross-platform data fusion, and sentiment shift were selectively removed from the pipeline while keeping the remaining system unchanged. This approach helps evaluate the relative importance of each component in the overall trend detection process. By observing the system behavior under these modified configurations, the study highlights how each module contributes to generating stable and reliable trend signals within the framework.

### Limitations

While the proposed sentiment-driven, multi-platform trend detection framework demonstrates practical value for early identification of emerging business signals, several limitations should be acknowledged.

• First, the methodology relies on the availability and accessibility of social media data through platform APIs and automated workflows. Changes in API policies, rate limits, or data access restrictions can affect data coverage and continuity over time. As a result, certain platforms or content types may be underrepresented during specific periods, potentially influencing trend visibility.
• Second, although the framework integrates data from multiple platforms, it remains dependent on textual representations derived from user-generated content. Speech-to-text conversion enables inclusion of audio and video posts; however, transcription inaccuracies, background noise, or language ambiguity may introduce noise into the dataset. Subtle contextual cues such as sarcasm, humor, or visual symbolism may not always be fully captured through text-based processing.
• Third, sentiment classification, even with a hybrid Transformer-lexicon approach, is inherently probabilistic. Informal language, evolving slang, and culturally specific expressions can still lead to misclassification in edge cases. While engagement-aware sentiment weighting improves signal quality, it does not entirely eliminate ambiguity in subjective interpretations.
• Fourth, the TrendScore formulation uses predefined weighting parameters to balance momentum, engagement, sentiment shift, and cross-platform presence. Although these weights are designed for general SMB scenarios, they may not optimally reflect priorities across all industries or regional markets without further domain-specific tuning.
• Finally, the validation of this work focuses on methodological soundness and practical relevance rather than large-scale quantitative benchmarking. While the framework demonstrates earlier and more interpretable trend detection than manual monitoring, future work could include controlled comparisons against commercial analytics platforms or long-term predictive accuracy studies.

Despite these limitations, the framework provides a transparent, extensible, and SMB-friendly foundation for transforming social media signals into actionable business intelligence.

### Significance and practical implications

The proposed framework offers SMBs an interpretable and reproducible solution to the opaque enterprise-grade analytics systems. The combination of cross-platform fusion, hybrid sentiment modeling, and explainable TrendScore computation makes it possible to identify early business opportunities and risks without the need for data science expertise. This approach fills the gap between advanced NLP research and practical SMB implementation.

### Ethics statements

This study utilizes only publicly available social media data and does not collect or store personally identifiable information. All data collection and processing comply with the usage policies of the respective platforms. The analysis is conducted at an aggregated level to prevent individual identification, ensuring ethical handling of user-generated content.

### CRediT author statement

Dipali Baviskar: Conceptualization, Supervision, Methodology. Harsh Halwai: Conceptualization, Implementation, Data processing. Anand Patekar: NLP modeling, Trend analysis. Anoushka Kachewar: Visualization, Validation, Documentation.

## Declaration of competing interest

The authors declare that they have no known competing financial interests or personal relationships that could have appeared to influence the work reported in this paper.

## Data Availability

No data was used for the research described in the article.
